# Mesenchymal stem cell-educated macrophages

**DOI:** 10.1186/2047-1440-1-12

**Published:** 2012-09-28

**Authors:** Elke Eggenhofer, Martin J Hoogduijn

**Affiliations:** 1 Department of Surgery, University Medical Center of Regensburg, Franz-Josef-Strauss Allee 11, Regensburg, Germany; 2 Department of Internal Medicine, Erasmus Medical Center, Rotterdam, The Netherlands

## Abstract

Mesenchymal stem cells (MSC) mediate their immunosuppressive effects via a variety of mechanisms. One of these mechanisms involves the induction of macrophages with immunomodulatory capacities. This effect of MSC may be exploited when MSC are used as a cell therapeutic product. Furthermore, MSC are resident in tissues where they may locally target infiltrating macrophages to adapt more regulatory properties. The present review discusses the interaction between MSC and macrophages, the induction of MSC-educated macrophages, how these cells position between other immune regulatory cells, and how they may be used in the clinic.

## Introduction

Mesenchymal stem cells (MSC) are stromal cells with potent regenerative and immunomodulatory properties [1,2]. They are found in multiple tissues, including bone marrow and adipose tissue [3] and are relatively easy to isolate and expand in culture. Their capacity to differentiate into multiple cellular lineages and their trophic effects on other progenitor cells has initiated interest in the use of these cells for regenerative therapy [4-6]. However, analysis of the mechanisms involved in the reparative effects of MSC indicates that many of these effects may in fact relate to the immunomodulatory properties of MSC. It has been demonstrated that MSC ameliorate acute graft *vs.* host disease [7,8], reduce the progression of kidney fibrosis by modulation of the early inflammatory response after injury [9,10], and in experimental models show promise as a therapeutic treatment of immunological diseases including arthritis [11], hepatitis [12], and organ transplantation [13,14].

MSC have the capacity to modulate the immune system via a plethora of mechanisms. They secrete anti-inflammatory factors such as TGF-β and hepatocyte growth factor [2], they inhibit lymphocyte proliferation via the expression of indoleamine 2, 3-dioxygenase (IDO) [15], and they express inhibitory co-stimulatory molecules such as programmed death ligand 1 (PD-L1) and TNF-related apoptosis-inducing ligand (TRAIL) [16,17]. In addition, MSC modulate the immune system via indirect mechanisms by inducing immune cells to adapt a regulatory function. MSC induce regulatory T cells *in vitro* and *in vivo*[14,18] and affect the differentiation and function of dendritic cells [19]. In recent years it has become clear that MSC also regulate the function of macrophages. Co-culture with MSC induces macrophages to adapt an enhanced regulatory phenotype via expression of increased levels of IL-10, reduced levels of TNF-α and IL-12, low co-stimulatory molecule CD86 and HLA class II while showing higher phagocytic activity [20,21]. This effect of MSC is at least partially mediated by soluble mechanisms and prostaglandin E2 (PGE-2) has been indicated to be one of the factors involved [21].

The question is whether the *in vitro* effects of MSC on macrophages are operational *in vivo*. There is evidence that MSC infusion leads to increased levels of regulatory type monocytes/macrophages in the circulation [22]. This effect is accompanied by an increase in the abundance of regulatory macrophages present within inflamed tissue [22]. Furthermore, it has been shown that locally administered MSC attract macrophages and turn them into a regulatory phenotype [21,23]. Thus *in vivo* administered MSC appear to have a similar effect on macrophages as their *in vitro* counterparts. The mechanisms involved, however, may be very different. There is accumulating evidence that, after administration, MSC are short-lived (Eggenhofer *et al*., in press). As transiently present MSC may themselves be incapable of secreting sufficient regulatory macrophages inducing factors, additional mechanisms may be important. Indeed, it has been demonstrated that the phagocytosis of dead MSC by macrophages induces them to adapt a more regenerative and immunomodulatory function [24]. This indicates that administered MSC may modulate macrophages function through initiating phagocytosis, while resident MSC that are around for a much longer period of time may modulate macrophages via the secretion of immunomodulatory factors and expression of cell surface molecules. In this respect it is interesting that throughout all tissues MSC are virtually ubiquitous. Tissue-resident MSC have the full capacity to locally induce regulatory macrophages. Studies into the influence of MSC on macrophages behavior are therefore relevant to assessments of MSC as a cell therapeutic product and also to examine the potentially exploitable effects of tissue-resident MSC.

### Generation of MSC-educated macrophages (MSC-Mo)

In the past, research to the immunomodulatory effect of MSC focused on the interactions of MSC with T-lymphocytes, B-lymphocytes, NK cells, and dendritic cells, but recently the effect of MSC on the cells of monocytic lineages, specifically macrophages, has attracted increasing attention. It is well known that MSC can generate an immunoregulatory type of macrophages *in vivo*[23]. Furthermore, it has been shown that MSC can also induce a regulatory macrophage population *in vitro*[20,21].

Recent studies have demonstrated the potential of MSC to educate macrophages to adapt an anti-inflammatory/immune suppressive phenotype. A number of studies allowed direct contact between MSC and macrophages *in vitro*[20,21]. However, other experiments have indicated that MSC can modulate macrophages via soluble factors in a transwell system [20].

**Figure 1:**
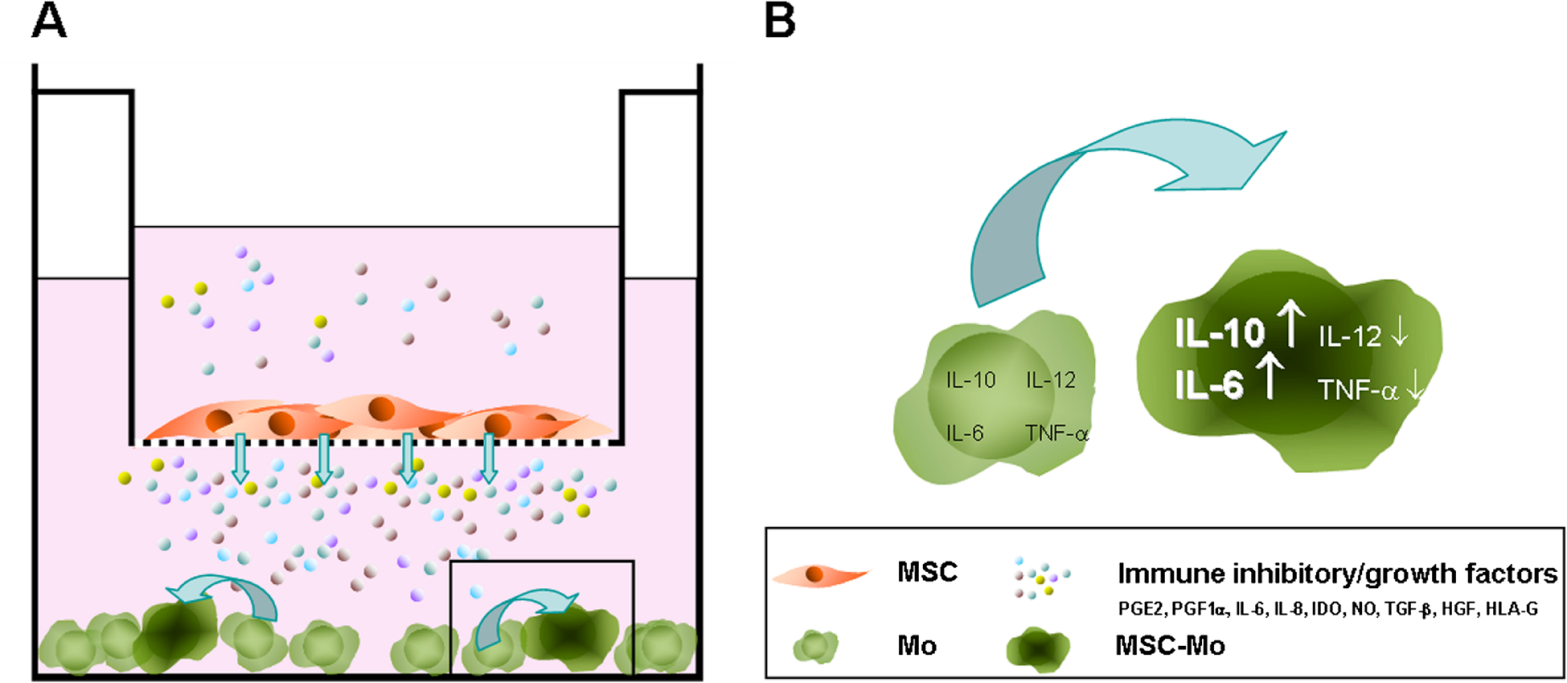
**Generation of MSC-educated macrophages (MSC-Mo). **(**A**) Co-cultivation of MSC and resting macrophages in a transwell cell culture system. Pores with a size of 0.4 μm allow exchange of MSC-produced soluble factors from upper chamber (MSC) to lower chamber (Mo). (**B**) Schematic overview of Mo to MSC-Mo transition induced by immunomodulatory (also in the figure) and growth factors released by MSC. Arrows next to cytokines show up- (↑) or down- (↓) regulation in MSC-Mo.

After co-culturing with MSC, peripheral blood monocytes derived macrophages (Mo) can be described as a novel type of alternatively activated macrophages (MSC-Mo) [20]. These MSC-Mo remain adherent to plastic and keep a Mo morphology. However, an increased number of Mo can be observed due to the trophic factors secreted by MSC (Figure 1A). They express higher levels of CD206, which is known to be a marker of alternatively activated macrophages and found on other types of anti-inflammatory macrophages as well [25]. Functionally, MSC-Mo display higher phagocytic activity compared to Mo. Moreover, these cells show increased production of anti-inflammatory IL-10 and IL-6, while their production of pro-inflammatory cytokines like IL-12 and TNFα is decreased (Figure 1B). Typically, alternatively activated macrophages are known to promote Th2 type of responses and secrete less pro-inflammatory cytokines (P. Riquelme: The ONE Study workshop 2012). However, these macrophages retain high levels of inflammatory cytokine production, such as TNF-α and IL-6 [26]. Based on those findings, MSC-Mo (IL-10high, IL-12low, IL-6high, and TNF-αlow) are defined as a novel type of alternatively activated population of macrophages distinct from previously reported macrophages [20].

### Application of MSC-Mo in the clinic

To date, MSC from adipose tissue or bone-marrow are used in several clinical trials in the treatment of a variety of clinical conditions such as graft *vs.* host disease [7,8], myocardial infarction [27], ischemic stroke [28], Crohn’s disease [29,30], diabetes mellitus [31], and acute graft rejection in organ transplantation [32,33]. These ostensibly dissimilar clinical conditions share the role of inflammation in their pathogenesis. Macrophages play a crucial role in not only the initiation but also the continuation of inflammatory processes. To activate macrophages in an alternative way by MSC may reduce inflammation and could modulate immune responses, which is of great therapeutic interest. Many groups and regulatory agencies favor the use of autologous cell therapy to avoid immune recognition. However, isolation, cultivation, and expansion of MSC to a clinical relevant dose normally can require several weeks and is not compatible with the treatment schedules of many conditions, particularly in the case of organ transplantation. Generation of MSC-Mo could be achieved by collection of monocytes through leukapheresis, followed by co-cultivation with a universal source of third party MSCs. Using this strategy a sufficient amount of autologous MSC-Mo could be prepared within a few days in a simple and clinical feasible fashion.

**Figure 2:**
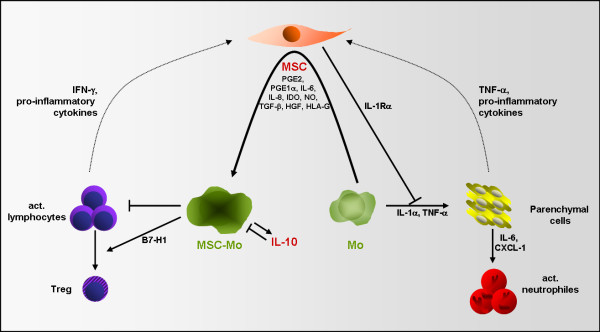
**Role of MSC in macrophage-mediated immune regulation *in situ*. **MSC modulate resting macrophages to adapt a regulatory phenotype by production of immunomodulatory and growth factors. This effect of MSC is enhanced by pro-inflammatory cytokines, released by activated immune cells and surrounding parenchymal cells. MSC thereby block Mo-mediated activation of parenchymal cells and decrease cellular immune response by generation of Tregs.

However, (pre-)clinical proof of concept studies with MSC-Mo have yet to be conducted and many questions remain concerning dosage and timing especially in the context of solid organ transplantation. Furthermore, a broader understanding of the potential role of MSC *in situ* and their influence on the generation of MSC-Mo could lead to the development of therapy whereby MSC are used as inducers of regulatory Mo *in vivo*. MSC reside in almost all tissue and will encounter infiltrating Mo in case of inflammation (Figure 2). MSC may thus locally affect the function of Mo and be capable of modulating immune responses in tissues. New therapies may therefore be designed that target tissue MSC to become activated (for example, mimicking local inflammation to create pro-inflammatory micromilieu by injection of pro-inflammatory cytokines) in order to stimulate infiltrating Mo to adapt a regulatory phenotype and function.

### Positioning of MSC-Mo between immune regulatory cells

MSC-Mo belong to a class of myeloid-derived suppressor cells. These cells share a myeloid origin and immune regulatory capacity, but are otherwise a heterogeneous population of cells. They are induced from immature myeloid cells by factors that are produced during acute or chronic tissue injury or disease [34]. One of these factors, PGE-2, induces myeloid-derived suppressor cells from dendritic cells [35] ([36] N. Obermayer: The ONE Study workshop 2012). Similarly, PGE-2 plays a role in the induction of MSC-Mo [21]. The production of PGE-2 by MSC increases under inflammatory conditions [37], suggesting an increased efficacy of MSC to induce regulatory Mo under conditions of injury. IL-10 is another factor associated with the generation of myeloid suppressor cells. It is a potent inducer of tolerogenic IL-10 producing dendritic cells [38], but it is doubtful whether it is involved in the induction of MSC-Mo. There is controversy whether MSC secrete IL-10 but in our hands levels of IL-10 secreted by MSC are neglectable [39]. Furthermore, IFN-γ, crucial for the formation of regulatory Mo [40] ([41] P. Riquelme and J. Hutchinson: The ONE Study workshop 2012), does not seem to be involved in the generation of MSC-Mo as MSC hardly secrete this cytokine [39,42]. MSC do secrete, however, a diversity of other factors of which several may play a role in the induction of MSC-Mo.

Functionally, MSC-Mo resemble several other types of myeloid-derived suppressor cells in some respects. Most of these cell types secrete IL-10 while their expression of pro-inflammatory cytokines like IL-6, TNF-α, and IL-12 is reduced. The expression profile of adhesion molecules and the migratory properties of MSC-Mo are not yet elucidated, however. MSC-Mo may differ considerably from non-adherent myeloid-derived suppressor cells in this respect ([43] C. Macedo: The ONE Study workshop 2012). *In vivo*, MSC-Mo are formed in tissues they have infiltrated and where they are exposed to a cocktail of cytokines provided by the stroma. Several types of myeloid-derived suppressor cells that are induced by a single or a small panel of cytokines may therefore come together in the MSC-Mo. Better characterization of the secretory profile of the stroma and the migration patterns of macrophages after encounter with the stroma will reveal more details on these interesting and potentially clinically useful cells.

## Conclusions

MSC provide macrophages with signals that stimulate conversion to a regulatory phenotype. There are two conceivable applications for the generation of MSC-Mo, the first by inducing MSC-Mo through MSC therapy, and the second by the induction of MSC-Mo by tissue-resident MSC. Pre-clinical and clinical studies that aim to use or target MSC in organ transplantation should consider that MSC-Mo may have a vital role in mediating the effects of MSC.

## Competing interests

The authors declare that they have no competing interests.

## Authors’ contributions

EE and MJH wrote the manuscript. Both authors read and approved the final manuscript.
